# Virtual family physician care during COVID-19: a mixed methods study using health administrative data and qualitative interviews

**DOI:** 10.1186/s12875-022-01902-9

**Published:** 2022-11-25

**Authors:** Bridget L. Ryan, Judith Belle Brown, Thomas R. Freeman, Lucie Richard, Moira Stewart, Leslie Meredith, Yun-Hee Choi, Jennifer Wei He, Sonny Cejic, Keith Thompson, Sonja Reichert, Salimah Z. Shariff, Richard Booth, Amanda L. Terry, Maria Mathews

**Affiliations:** 1 Western University, London, Canada; 2 ICES Western, London, Canada

**Keywords:** Family physicians, Virtual care, COVID-19, Pandemic, Primary health care, eHealth, Mixed methods

## Abstract

**Background:**

The onset of the COVID-19 pandemic necessitated a rapid shift in primary health care from predominantly in-person to high volumes of virtual care. The pandemic afforded the opportunity to conduct a deep regional examination of virtual care by family physicians in London and Middlesex County, Ontario, Canada that would inform the foundation for virtual care in our region post-pandemic. Objectives: (1) to determine volumes of in-person and virtual family physicians visits and characteristics of the family physicians and patients using them during the early COVID-19 pandemic; (2) to determine how virtual visit volumes changed over the pandemic, compared to in-person; and (3) to explore family physicians’ experience in virtual visit adoption and implementation.

**Methods:**

We conducted a concurrent mixed-methods study of family physicians from March to October 2020. The quantitative component examined mean weekly number of total, in-person and virtual visits using health administrative data. Differences in outcomes according to physician and practice characteristics for pandemic periods were compared to pre-pandemic. The qualitative study employed Constructivist Grounded Theory, conducting semi-structured family physicians interviews; analyzing data iteratively using constant comparative analysis. We mapped themes from the qualitative analysis to quantitative findings.

**Results:**

Initial volumes of patients decreased, driven by fewer in-person visits. Virtual visit volumes increased dramatically; family physicians described using telephone almost entirely. Rural family physicians reported video connectivity issues. By early second wave, total family physician visit volume returned to pre-pandemic volumes. In-person visits increased substantially; family physicians reported this happened because previously scarce personal protective equipment became available. Patients seen during the pandemic were older, sicker, and more materially deprived.

**Conclusion:**

These results can inform the future of virtual family physician care including the importance of continued virtual care compensation, the need for equitable family physician payment models, and the need to attend to equity for vulnerable patients. Given the move to virtual care was primarily a move to telephone care, the modality of care delivery that is acceptable to both family physicians and their patients must be considered.

**Supplementary Information:**

The online version contains supplementary material available at 10.1186/s12875-022-01902-9.

## Background

The onset of the COVID-19 pandemic generated a rapid shift in primary health care in Ontario, Canada from predominantly in-person care to high volumes of virtual care [1]. Prior to the pandemic, virtual care visits were uncommon with only 10% of Ontarians reporting having had a virtual visit with a physician; despite 69% of Canadians indicating they would choose virtual health visits if available to them [2].

Pre-pandemic, the only insurable option available to physicians for virtual health care services was provided through the Ontario Telemedicine Network (OTN), a secure virtual video platform [3, 4]. There was modest uptake of OTN among family physicians [5, 6]. In response to the pandemic, on March 14, 2020, the Ontario Ministry of Health instituted billing codes for virtual care, which permitted physicians to bill the Ontario Health Insurance Program (OHIP) for virtual synchronous patient interactions that could include telephone and video channels, making these visits insurable [7]. These billing codes were available to family physicians (FPs) practicing in all practice models. Practice models in Ontario include traditional fee-for-service where FPs bills OHIP for each service provided to a patient according to a payment schedule [8], enhanced fee-for-service where FPs are compensated using predominantly fee-for-service but with specific premiums and bonuses for patient enrolment, capitation-based models where FPs are compensated primarily through capitation [9].

The province of Ontario, with a population of approximately 14.7 million people [10], reported the first case of the novel coronavirus 2019 (COVID-19) cases in January 2020 [11, 12], and on March 17, 2020, the Government of Ontario declared a state of emergency to curb the impact of the pandemic [13].

The Middlesex-London Public Health Unit is located in the southwestern region of the province and includes the City of London and the County of Middlesex. This region is situated halfway between the US-Canada border and the Greater Toronto Area (Ontario’s most densely populated region). Covering an estimated population of 510,609, this region includes both urban and rural settings [14]. London is used often as a testing ground for new products because it is considered representative of the rest of Canada [15]. This region recorded the third case of COVID-19 identified in Ontario [16] and there were 1160 COVID-19 cases (case rate 231 per 100,000) and 60 deaths (death rate 12 per 100,000) between March and October of 2020 [17]. This region’s case and death rates were lower than Ontario (524 cases per 100,000 and 21.4 deaths per 100,000) and Toronto (capital of Ontario with population of approximately 3 million) (916 cases per 100,000 and 45.4 deaths per 100,000) [17].

The pandemic afforded the opportunity to study the rapid uptake and delivery of virtual care by FPs, albeit in a time of crisis. Through this study, we sought to understand the experiences of FPs in London and Middlesex County, concerning virtual care during the early pandemic, as one essential step in building a foundation for virtual care in our region post-pandemic. Given the diversity of the pandemic experience across Ontario, as it was in other parts of the world, a deep examination of regional experience is important. Our specific objectives were: (1) to determine volumes of in-person and virtual FP visits and the characteristics of the FPs and patients using them during the early COVID-19 pandemic; (2) to determine how the volume of virtual visits changed over the course of the pandemic, compared to in-person visits; and (3) to explore the FPs’ experience in adoption and implementation of virtual visits by FPs.

## Methods

### Design and setting

This was a concurrent mixed-methods study [18] of FPs practising family medicine in London and Middlesex County, where approximately 84% of the population live in London [19, 20]. It is estimated that 89% of the Middlesex-London population is attached to a family physician, meaning they have access to primary care through a family physician [21]. The period of the study, March to October 2020, corresponded to the first and second waves of the COVID-19 pandemic in this region [11, 13, 17-Ontario weekly case counts]. The quantitative component was a secondary analysis using health administrative (HA) data at ICES [22]. ICES is an independent, non-profit research institute whose legal status under Ontario’s health information privacy law allows it to collect and analyze health care and demographic data, without consent, for health system evaluation and improvement. All residents of Ontario obtain their medical and hospital health care services from a government-administered, single-payer insurance system that maintains a comprehensive electronic provider reimbursement claims database. As such, data from ICES constitute population-level information. The qualitative study consisted of semi-structured interviews and employed Constructivist Grounded Theory (CGT) [23].

### Quantitative study methods

#### Cohort and data

Additional file 1: Appendix 1 describes the datasets that were linked and used in the HA analysis which were: Canadian Institute for Health Information Discharge Abstract Database (DAD), Chronic Obstructive Pulmonary Disease (COPD), Congestive Heart Failure (CHF), Institute for Clinical Evaluative Sciences Physician Database (IPDB), National Ambulatory Care Reporting System (NACRS), Ontario Asthma Dataset (ASTHMA), Ontario Crohn’s and Colitis Cohort Dataset (OCCC), Ontario Dementia Database (DEMENTIA), Ontario Diabetes dataset (ODD), Ontario Drug Benefit Claims (ODB), Ontario Health Insurance Plan Claims Database (OHIP), Ontario HIV Dataset (HIV), Ontario Hypertension Dataset (HYPER), Ontario Marginalization Index (ONMARG), Ontario Mental Health Reporting System (OMHRS), Ontario Rheumatoid Arthritis Database (ORAD), Primary Care Population (PCPOP), Registered Persons Database (RPDB), and Same Day Surgery Database (SDS). These datasets were linked using unique encoded identifiers and analyzed at ICES.

##### Cohort creation

We identified FPs practicing in London and Middlesex County, defined here as the region census division (CD 3539), as of March 14 2020 [19]. These FPs were identified from the ICES Physician Database (IPDB), which is a cumulative database that contains all FPs including those retired and deceased. Therefore, to identify active FPs, we excluded FPs: without billings in OHIP in the 12 months prior to March 14, 2020, who had less than 10% of their billings as family medicine billings (to exclude those who primarily practice as non-FPs), and who were in the bottom 10% of absolute number of billings (to exclude those who were on parental leave, had administrative rather than clinical roles, or who were likely retired or mostly-retired).

##### Covariates

For each of the physicians, we identified physician age category (50+ years/< 50 years), physician sex (male/female), physician experience (years in practice: < 5 years, 5- < 10 years, 10+ years), practice location (rural/urban), practice model (fee for service, enrolment model), and physician workload (less than 1.0 full-time equivalent (FTE), 1 FTE). Practice characteristics refer to the profile of the patients that were seen (either in-person or virtually) at each practice during the study period, aggregated as means across all practices. Patient profile variables that were aggregated included: average age and proportion of age groups of patients; proportion of patients by sex; proportion of patients at each level of neighbourhood income; proportion of patients at each level of Ontario marginalization material deprivation score [24]; proportion of patients that identified as having multimorbidity [25]; and proportion of patients with various chronic conditions [25].

##### Outcomes

We identified for each physician: mean weekly number of total, in-person and virtual visits across each period of interest. For total visits and in-person visits, we characterized the outcome as a ratio of visits during the pandemic period divided by baseline visits, while virtual visits, having no baseline against which to compare, were modelled as a proportion of total visits.

##### Timeframe

Characteristics and outcomes of the physicians and their practices were identified at four times: at baseline (March 14, 2019 to March 13, 2020), during the early first wave of the pandemic (March 14 to May 10, 2020); during the lull between the first and second waves (May 11 to September 17, 2020); and the early portion of the second wave (September 18 to the end of our observation period, October 31, 2020).

#### Quantitative data analysis

All analyses were conducted using SAS version 9.4 (SAS Institute, Cary, NC). Characteristics were reported for the FPs and their practices (characteristics of patients seen in those practices during the study period) in each of the pre-pandemic and pandemic periods. Differences in characteristics for each pandemic period compared to baseline were determined using one-way ANOVA or chi-squared test as applicable. Mean weekly total, in-person and virtual visits were visually represented in a trend figure across the pre-pandemic and pandemic periods. An interrupted time series using segmented regression (reported as an appendix) examined statistically significant trends across different periods during the pandemic.

We further fit multivariable linear regression models to evaluate the association between FP characteristics (age and sex) and practice characteristics (practice location and model, full-time equivalent status; % practice female patients and % practice patients with multimorbidity) and the outcomes. Assumptions for linear regression were tested.

### Qualitative study methods

#### Recruitment and data collection

Potential FP participants were identified through publicly available lists and by study investigators and recruited through email. Potential participants were provided a letter of information and signed a consent form to participate. We purposefully recruited FPs, ensuring variation in age, sex, family practice model, and location (urban/rural).

The Principal Investigator (BLR) conducted all semi-structured interviews, which were 45 to 60 minutes in length using either Zoom or telephone. Interviews were audio-recorded using an external recording device and transcribed verbatim. Interviews took place from July 2020 to February 2021 and participants were asked to reflect back on the period from March 14, 2020 to the time of the interview, to capture the period used for the quantitative component of the study as closely as possible.

#### Qualitative data analysis

Three members of the research team (BLR, JBB, TF) conducted the analysis using Constructivist Grounded Theory methods [22]. We conducted the analysis iteratively and alongside data collection, supplementing the semi-structured interview guide as new themes emerged from the data. We first coded each transcript independently line by line. We then conducted focussed coding as a team, consolidating codes and deciding which codes best represented the data. We used constant comparative analysis, revisiting older data when new concepts emerged to refine our understanding. One author (JWH) input the codes into NVivo software [26]. Finally, we employed theoretical coding where we sought relationships among the codes. We made extensive memos of possible intersections among themes as they developed, areas to revisit and new themes we probed in subsequent interviews. As well, we identified any gaps in our sample of participants and used theoretical sampling to ensure a diverse sample of family physicians [23]. After we had reached data sufficiency [27], we reviewed a report of all codes, consolidated themes and developed the narrative. We enhanced trustworthiness throughout the data collection and analysis by employing reflexivity [23] to consider how we influence the research process, given our own disciplines (BLR-Epidemiology, JBB-Social Work, TF-Family Medicine) and perspectives.

### Integration of quantitative and qualitative data

We brought together the analyses from the quantitative and qualitative data collections by mapping the themes from the qualitative analysis to the quantitative findings. We looked for consistent and dissonant messages as well as instances where one or the other of the methods could provide information unique to that data collection method. We present the results as one narrative according to broad themes identified.

## Results

**Fig. 1:**
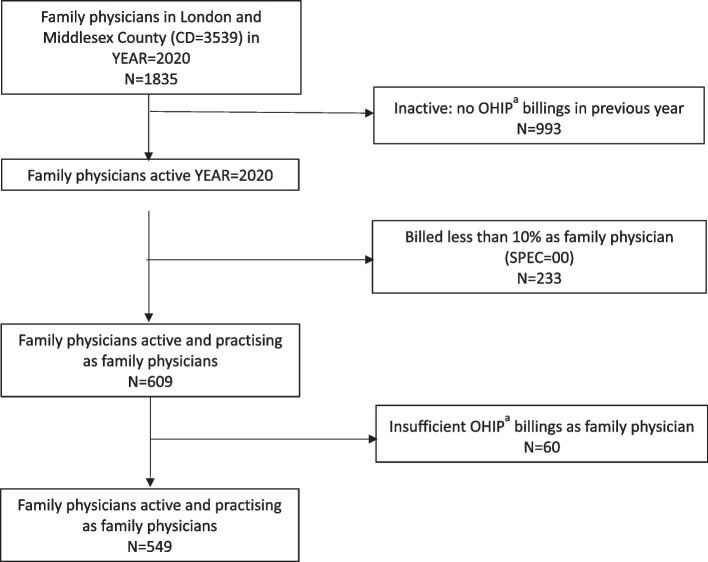
Quantitative Study Hierarchy – Inclusion and Exclusion ^**a**^ OHIP = Ontario Health Insurance Plan

Figure 1 reports the creation of the study cohort for the quantitative study component. There was a decrease in the number of FPs seeing patients for each of the three pandemic waves compared to pre-pandemic; however, there were no significant differences in the characteristics of these FPs compared to pre-pandemic values (Table 1). Table 2 reports the characteristics of the 17 FPs interviewed for the study’s qualitative component.

**Table 1:** Characteristics of London and Middlesex County, Ontario Family Physicians Before and During COVID-19 Pandemic (Column %)

	Pre-Pandemic^**a**^	Early 1st Wave^**a**^	Mid-Late 1st Wave^**a**^	Early 2nd Wave^**a**^	Compared to Pre-pandemic ***p***-value^**b**^		
**Family Physician Characteristics**	***N*** **= 549**	***N*** **= 515**	***N*** **= 520**	***N*** **= 500**	**Early 1st Wave**^**a**^	**Mid-Late 1st Wave**^**a**^	**Early 2nd Wave**^**a**^
Age							
18–49 years, N (%)	288 (52.5%)	272 (52.8%)	274 (52.7%)	266 (53.2%)	0.907	0.939	0.81
50+ years, N (%)	261 (47.5%)	243 (47.2%)	246 (47.3%)	234 (46.8%)			
Missing, N (%)	31 (5.6%)	29 (5.6%)	30 (5.8%)	28 (5.6%)			
Sex							
Female, N (%)	265 (48.3%)	245 (47.6%)	251 (48.3%)	242 (48.4%)	0.972	0.996	0.999
Male, N (%)	253 (46.1%)	241 (46.8%)	239 (46.0%)	230 (46.0%)			
Missing, N (%)	31 (5.6%)	29 (5.6%)	30 (5.8%)	28 (5.6%)			
Age by Sex							
Female 18–49 years, N (%)	148 (27.0%)	136 (26.4%)	137 (26.3%)	133 (26.6%)	0.997	0.994	0.985
Female 50+ years, N (%)	117 (21.3%)	109 (21.2%)	114 (21.9%)	109 (21.8%)			
Male 18–49 years, N (%)	109 (19.9%)	107 (20.8%)	107 (20.6%)	105 (21.0%)			
Male 50+ years, N (%)	144 (26.2%)	134 (26.0%)	132 (25.4%)	125 (25.0%)			
Unknown, N (%)	31 (5.6%)	29 (5.6%)	30 (5.8%)	28 (5.6%)			
Rurality							
Urban, N (%)	503 (91.6%)	471 (91.5%)	475 (91.3%)	458 (91.6%)	0.984	0.984	0.997
Rural, N (%)	15 (2.7%)	15 (2.9%)	15 (2.9%)	14 (2.8%)			
Missing, N (%)	31 (5.6%)	29 (5.6%)	30 (5.8%)	28 (5.6%)			
Practice model							
FFS/FFS enhanced, N (%)	322 (58.7%)	291 (56.5%)	295 (56.7%)	282 (56.4%)	0.479	0.525	0.461
Other, N (%)	227 (41.3%)	224 (43.5%)	225 (43.3%)	218 (43.6%)			
Full-time equivalent, N (%)	326 (59.4%)	156 (30.3%)	241 (46.3%)	305 (61.0%)	**<.001**	**<.001**	0.593

^a^Pre-pandemic defined as 12 months prior to March 14, 2020, Early 1st Wave defined as March 14–May 10, 2020, Mid-Late 1st Wave defined as May 11–September 17, 2020, Early 2nd Wave defined as September 18–October 31, 2020

^b^Chi-square test was used

**Table 2:** Characteristics of London and Middlesex County, Ontario family physicians interviewed for qualitative component (*n* = 17)

Family Physician Characteristic	n (%)
Age by Sex	
Female 18–49 years	2 (12%)
Female 50+ years	5 (29%)
Male 18–49 years	5 (29%)
Male 50+ years	5 (29%)
Practice model	
Fee for service/Enhanced fee for service	3 (18%)
Other	14 (82%)
Location	
London	14 (82%)
Rural outside London	3 (18%)

We organized the Results section by headings that represent the Quantitative Results (which arose from analyzing the health administrative data) and the Qualitative Themes (from our constant comparative and theoretical coding analysis). Within each of these sections, connections are made between the quantitative results and the qualitative findings. The qualitative themes were: Access to care; Transitions in Providing Care during COVID-19 (sub-themes: Always being open, Initial volumes of visits dropped, Reluctance to meet in person, and Rapid move to virtual care); and Inequity of Payment Models.

### Access to care

During the pandemic waves, FP practices saw a greater proportion of patients 65 years of age and older, with a lower proportion of male patients and a higher proportion of patients with multimorbidity. The proportion of the most materially deprived patients was higher than pre-pandemic during the first wave of the pandemic (Table 3).

**Table 3:** Aggregated practice characteristics^a^ London and Middlesex County, Ontario family physicians before and during COVID-19 pandemic (Column %)

Practice Characteristics^**a**^	Pre-Pandemic^**b**^	Early 1st Wave^**b**^	Mid-Late 1st Wave^**b**^	Early 2nd Wave^**b**^	Compared to Pre-pandemic ***p***-value^**c**^		
	***N*** = 549	***N*** = 515	***N*** = 520	***N*** = 500	Early 1st Wave^**b**^	Mid-Late 1st Wave^**b**^	Early 2nd Wave^**b**^
Age							
% < 65 years, mean ± SD	74.34 ± 15.32	70.41 ± 16.76	70.97 ± 16.45	67.89 ± 18.09	**<.001**	**<.001**	**<.001**
% > = 65 years, mean ± SD	25.66 ± 15.32	29.59 ± 16.76	29.03 ± 16.45	32.11 ± 18.09	**<.001**	**<.001**	**<.001**
Sex							
% Female, mean ± SD	57.78 ± 9.67	58.83 ± 11.76	59.18 ± 11.46	60.37 ± 11.99	0.11	**0.031**	**<.001**
% Male, mean ± SD	42.19 ± 9.67	40.80 ± 11.88	40.80 ± 11.45	39.60 ± 11.99	**0.036**	**0.032**	**<.001**
% Missing, mean ± SD	0.04 ± 0.04	0.37 ± 2.35	0.02 ± 0.09	0.03 ± 0.17	**<.001**	**0.011**	0.446
% Income quintile, mean ± SD							
1-lowest income	18.91 ± 7.62	20.77 ± 8.98	20.34 ± 8.74	19.89 ± 9.24	**<.001**	**0.004**	0.06
2	18.77 ± 5.10	20.01 ± 6.37	19.22 ± 5.66	19.49 ± 7.13	**<.001**	0.169	0.057
3	18.27 ± 4.19	18.73 ± 5.78	18.68 ± 4.90	18.33 ± 5.47	0.14	0.144	0.841
4	20.82 ± 6.00	20.66 ± 7.65	21.30 ± 7.55	21.00 ± 7.20	0.703	0.253	0.655
5-highest income	20.20 ± 9.07	18.62 ± 10.19	19.66 ± 9.42	20.43 ± 10.39	**0.008**	0.343	0.702
Missing	3.04 ± 7.04	1.22 ± 2.70	0.81 ± 1.17	0.85 ± 1.49	**<.001**	**<.001**	**<.001**
% Material deprivation, mean ± SD							
1-least deprived	26.08 ± 10.28	25.10 ± 11.20	26.09 ± 11.08	26.50 ± 11.71	0.133	0.999	0.541
2	18.58 ± 4.58	18.54 ± 6.56	18.73 ± 5.71	18.89 ± 6.13	0.895	0.639	0.354
3	13.14 ± 3.72	13.32 ± 5.04	13.31 ± 4.38	13.35 ± 4.98	0.497	0.5	0.441
4	16.99 ± 4.47	18.00 ± 5.95	17.97 ± 5.38	17.64 ± 6.72	**0.002**	**0.001**	0.062
5-most deprived	21.26 ± 9.57	22.85 ± 10.92	22.19 ± 10.36	21.90 ± 11.01	**0.012**	0.128	0.321
% Multimorbidity, mean ± SD	33.38 ± 17.02	40.66 ± 16.62	36.78 ± 15.84	38.11 ± 15.85	**<.001**	**<.001**	**<.001**
% Chronic Conditions, mean ± SD							
Arthritis	12.47 ± 6.20	14.33 ± 7.13	13.98 ± 7.97	14.66 ± 7.48	**<.001**	**<.001**	**<.001**
Cancer	6.36 ± 11.76	6.41 ± 9.52	6.05 ± 8.83	5.83 ± 7.20	0.936	0.625	0.385
Congestive heart failure	4.75 ± 5.50	5.36 ± 4.86	4.58 ± 4.41	4.80 ± 4.43	0.055	0.584	0.863
COPD^d^	10.95 ± 6.72	13.01 ± 7.14	12.05 ± 6.95	12.28 ± 6.86	**<.001**	**0.008**	**0.001**
Cardiovascular disease	6.73 ± 6.65	6.94 ± 5.14	6.49 ± 6.40	6.46 ± 6.36	0.566	0.551	0.508
Dementia	3.76 ± 7.90	4.62 ± 9.28	3.58 ± 7.84	3.97 ± 8.16	0.105	0.706	0.682
Diabetes	14.89 ± 9.22	18.97 ± 9.70	16.96 ± 9.10	17.45 ± 9.26	**<.001**	**<.001**	**<.001**
HIV	9.28 ± 8.24	10.70 ± 7.40	9.20 ± 6.91	9.32 ± 5.71	**0.003**	0.859	0.93
Hypertension	30.80 ± 13.97	36.98 ± 15.36	34.48 ± 14.64	35.92 ± 15.24	**<.001**	**<.001**	**<.001**
Kidney disease, chronic	2.54 ± 3.95	2.55 ± 2.89	2.06 ± 2.45	2.17 ± 2.61	0.954	**0.02**	0.08
Liver disease, chronic	2.78 ± 2.99	2.80 ± 2.81	2.41 ± 2.29	2.31 ± 2.33	0.893	**0.025**	**0.005**
Mood disorder	22.54 ± 13.53	26.85 ± 15.86	23.21 ± 13.57	23.44 ± 14.35	**<.001**	0.418	0.297
Osteoporosis	1.74 ± 1.47	1.91 ± 1.71	1.74 ± 1.44	2.02 ± 1.89	0.077	0.972	**0.008**
Stroke/TIA	2.19 ± 4.84	2.24 ± 3.53	1.76 ± 2.34	1.74 ± 1.98	0.851	0.068	0.053
Urinary incontinence	4.49 ± 2.72	5.31 ± 5.27	4.52 ± 2.60	4.45 ± 2.61	**0.001**	0.839	0.818

^a^Aggregated Practice Characteristics refers to the profile of the patients that were seen (either in-person or virtually) at each practice during the study period, aggregated as means across all practices

^b^Pre-pandemic defined as 12 months prior to March 14, 2020, Early 1st Wave defined as March 14–May 10, 2020, Mid-Late 1st Wave defined as May 11–September 17, 2020, Early 2nd Wave defined as September 18–October 31, 2020

^c^One-way ANOVA was used

^d^COPD=Chronic Obstructive Pulmonary Disease

These findings were consistent with the theme of “access to care” from the qualitative interviews. All FPs reported that virtual care improved access for many vulnerable patients, “the accessibility for a lot of patients is really improved”. (FP10, M, 37 years). Regarding older patients accessing virtual care, one FP said, “I think it’s a bit of a misnomer to think that elderly patients don’t use technology.” (FP11, M, 32 years). One FP expressed it was safer for older patients to be seen virtually, “… my 80-year-old doesn’t have to take a taxi out in the winter, walk on the ice and get up my stairs … It’s safer if they don’t really need to be examined.” (FP16, F, 52 years). Other FPs talked about how virtual care removed barriers for some patients; for example, patients who depend on public transportation: “These young moms with kids taking three buses because there’s only one bus route that actually goes past my door.” (FP6, M, 64 years). Participants also acknowledged how virtual care improved access for patients who have difficulty taking time off work: “… so long haul truck drivers in the States have done diabetic [virtual] visits with me from their cab.” (FP10, M, 37 years) and “… a lot of my patients who have jobs … it was a half a day off work [to come into office]” (FP6, M, 64 years).

In contrast to this overall positive perspective concerning improved access, some FPs who cared for vulnerable patients such as those who were experiencing homelessness, cautioned that for those patients, virtual care could be difficult, “a big issue … is patients [without housing] having access to virtual care. Even a telephone sometimes is tricky for them.” (FP7, F, 46 years).

The other barrier to access, raised by FPs in rural areas, was that FPs and their patients did not always have access to high-speed internet. This was especially problematic if an FP wanted to conduct a video rather than a telephone visit. “We had some technical difficulties with bandwidth, both for us [the FPs] and for them [the patients] … pictures really, really choppy … we switched to phone just because it turned out to be much easier to do” (FP3, F, 58 years).

### Volume and mode of FP visits and transitions in providing care during COVID-19

**Fig. 2:**
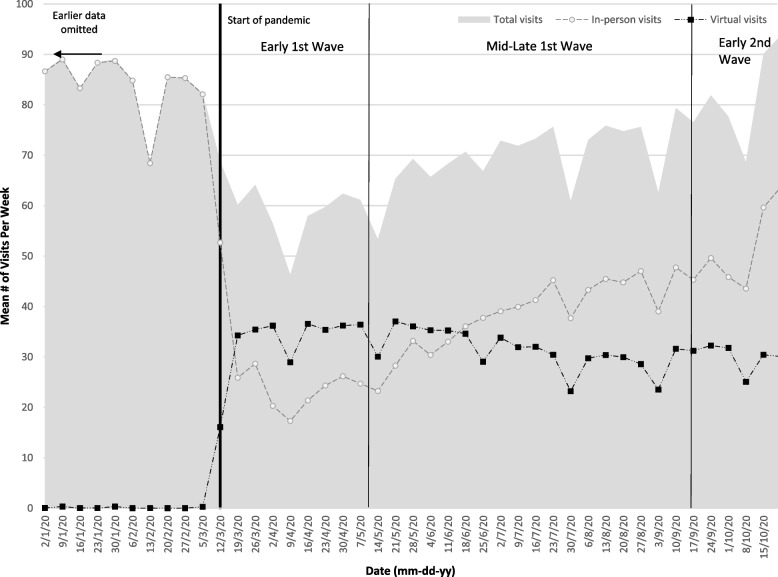
Mean Number of Weekly Visits for London and Middlesex County, Ontario Family Physicians Before and During the COVID-19 Pandemic^**a**^ ^**a**^ Pre-pandemic defined as 12 months prior to March 14, 2020, Early 1st Wave defined as March 14–May 10, 2020, Mid-Late 1st Wave defined as May 11–September 17, 2020, Early 2nd Wave defined as September 18–October 31, 2020

Table 4 reports, for FPs who provided services, the mean weekly FP visits (total, in-person and virtual) pre-pandemic and then during the three pandemic periods of this study. Figure 2 visually describes the change in volume of FP visits over the course of the study period. Additional file 1: Appendix 2 reports the interrupted time series that describes statistically what is seen in the graph in Fig. 2. The volume of FP in-person visits dropped 72.6% within 2 weeks of March 14 resulting in an overall volume drop of 35.8% because the in-person decrease was offset by the increase in virtual visits from essentially zero per week (0.08 visits/week) to 29.4 visits per week. Within the two-week period following March 14, virtual visits comprised 56.3% of total visits. By June 15, in-person visits started to increase and overtook virtual visits. Virtual visits decreased slightly but plateaued. Total FP visits returned to pre-pandemic levels by October 2020.

**Table 4:** Mean number of weekly visits for London and Middlesex County, Ontario family physicians before and during the COVID-19 pandemic

	Pre-Pandemic^**a**^	Early 1st Wave^**a**^	Mid-Late 1st Wave^**a**^	Early 2nd Wave^**a**^
**WEEKLY FP VISITS**	***N*** **= 549**	***N*** **= 515**	***N*** **= 520**	***N*** **= 500**
In-person FP Visits				
Mean (SD)	79.68 (45.93)	21.87 (25.88)	35.16 (32.96)	47.86 (36.68)
Median (IQR)	70.65 (53.7–98.52)	14.37 (6.11–28.22)	27.52 (15.37–47.11)	40.67 (25.42–62.75)
% of Total visits	99.79%	43.67%	54.70%	63.65%
Virtual FP Visits				
Mean (SD)	0.08 (1.19)	29.36 (24.51)	28.72 (26.09)	27.78 (26.96)
Median (IQR)	0 (0–0)	28.11 (8.67–42.67)	25.08 (7–42.5)	23.25 (5.25–43.17)
% of Total Visits	0.21%	56.33%	45.30%	36.35%
TOTAL FP VISITS				
Mean (SD)	79.77 (45.89)	51.23 (31.59)	63.88 (39.48)	75 .64 (44.71)
Median (IQR)	70.72 (53.7–98.9)	46.2 (30–65.9)	57.9 (38.5–80.1)	71 (42.4–97.42)

^a^Pre-pandemic defined as 12 months prior to March 14, 2020, Early 1st Wave defined as March 14–May 10, 2020, Mid-Late 1st Wave defined as May 11–September 17, 2020, Early 2nd Wave defined as September 18–October 31, 2020

These findings were reflected in the qualitative theme concerning “transitions in providing care during COVID-19”. The FP interviews elaborated reasons for the trends we observed in Fig. 2. We identified a sub-theme of “always being open” with FPs indicating that, for the most part, they did not shut down their offices, “and if we couldn’t [do virtual] then we were available to do in-person visits from the get-go...” (FP7, F, 46 years). The sub-theme, “initial volumes of visits dropped” described the overall drop in visits in the early days of the pandemic, “I mean, the demand plummeted. I think we were seeing about 20 to 30% of our usual capacity – this is including virtual care...” (FP17, M, 44 years).

Regarding the decrease of in-person visits, the sub-theme, “reluctance to meet in person” described the reasons this happened, “Patients didn’t want to go out, they were all fearful.” (FP8, M, 65 years). Another FP indicated, “Everything needed to be done by telephone for patient safety and for staff safety.” (FP10, M, 37 years). An initial shortage of Personal Protective Equipment (PPE) prevented some FPs from seeing patients in-person, “So, I was seeing people but at a much lower volume... we really didn’t have PPE or anything “. (FP16, F, 52 years). Some FPs would direct patients with COVID-19 symptoms to places “where people had PPE and the equipment to deal with higher risk patients … then as we started seeing people in person, it was really as we started securing PPE.” (FP10, M, 37 years).

FPs talked about the “rapid move to virtual care”, which is congruent with the quick uptake of virtual care seen in Fig. 2; the FP interviews observed that this uptake was overwhelmingly through the telephone, “We moved everybody that we determined didn’t need to come in … and we started with phone visits exclusively …” (FP12, F, 59 years). While some FPs used OTN successfully, a number of FPs reported they and/or their patients found it problematic, “So OTN, we started using, but it’s very clumsy getting on to it. We have more recently used a [private vendor] video platform, which is easy because basically I go from my scheduler right into the video conference. Like, it’s seamless and patients, on their end, find it very easy.” (FP13, M, 55 years).

### FP and practice characteristics and inequity of payment models

Table 5 describes the association between FP and practice characteristics, across each pandemic period, with total, in-person, and virtual visits as a proportion of total visits, compared to pre-pandemic levels. Assumptions for linear regression were met. The overall estimates for the statistically significant characteristics were generally very small and inconsistent across the three pandemic periods. In at least one of the three pandemic periods, *mean weekly total FP visits*, compared to pre-pandemic levels, were higher for younger and full-time FPs, and FPs for whom higher percentages of their patients were female and had multimorbidity. *Mean weekly in-person visits*, compared to pre-pandemic baselines, were associated with being an older FP, practising in a fee-for-service practice model, and having a higher proportion of patients with multimorbidity. Compared to baseline, FPs had higher *mean weekly virtual visits as a proportion of their total visits* if they were older, in a capitation practice model, and had higher proportions of their patients who were female. FPs had a *lower proportion of virtual visits* if they had a higher percentage of patients with multimorbidity.

**Table 5:** Characteristics associated with Total, In-Person and Virtual Mean Weekly Family Physician Visits in London and Middlesex County, Ontario (*N* = 549)

***a. Linear Regression of Ratio of Pandemic to Pre-pandemic for Total Mean Weekly Visits (N = 549)***						
**Ratio of Pandemic to Pre-pandemic for Total Mean Weekly Visits**	**Early 1st Wave**^**a**^ **to Pre-pandemic**		**Mid-Late 1st Wave**^**a**^ **to Pre-pandemic**		**Early 2nd Wave**^**a**^ **to Pre-pandemic**	
**Characteristics at Baseline**^**b**^	**Estimate**	**95% CI**	**Estimate**	**95% CI**	**Estimate**	**95% CI**
FP age > 50 (ref = < 50 years)	−0.024	−0.077, 0.029	− 0.045	− 0.106, 0.015	**− 0.112**	**− 0.204, − 0.02**
FP Sex Male (ref = female)	− 0.052	− 0.118, 0.013	− 0.030	− 0.104, 0.044	− 0.034	− 0.147, 0.079
Practice location rural (ref = urban)	−0.033	− 0.191, 0.126	0.024	− 0.157, 0.204	− 0.014	−0.288, 0.26
FP age/sex/rurality unknown (ref = < 50 years, female, urban known)	0.034	−0.086, 0.154	**0.152**	**0.016, 0.289**	0.156	−0.052, 0.364
Practice model (ref = FFS/FFS enhanced^c^)	0.009	−0.048, 0.066	0.008	−0.057, 0.073	0.067	−0.032, 0.166
Full-time equivalent (ref = less than full-time)	**0.065**	**0.005, 0.125**	0.061	−0.007, 0.129	**0.116**	**0.013, 0.219**
Mean % female patients	**0.003**	**0, 0.007**	0.003	−0.001, 0.007	0.002	−0.004, 0.007
Mean % patients with multimorbidity^c^	**0.002**	**0.001, 0.004**	**0.003**	**0.001, 0.004**	0.000	−0.003, 0.003
***b. Linear Regression of Ratio of Pandemic to Pre-pandemic for In-Person Mean Weekly Visits (N = 549)***						
**Ratio of Pandemic to Pre-pandemic for In-Person Mean Weekly Visits**	**Early 1st Wave**^**a**^ **to Pre-pandemic**		**Mid-Late 1st Wave**^**a**^ **to Pre-pandemic**		**Ratio of Early 2nd Wave**^**a**^ **to Pre-pandemic**	
**Characteristics at Baseline**^**b**^	**Estimate**	**95% CI**	**Estimate**	**95% CI**	**Estimate**	**95% CI**
FP age > 50 (ref = < 50 years)	**−0.050**	**−0.087, − 0.013**	**−0.085**	**− 0.133, − 0.037**	**−0.107**	**− 0.182, − 0.032**
FP Sex Male (ref = female)	0.007	− 0.038, 0.052	0.002	− 0.057, 0.061	−0.006	− 0.099, 0.086
Practice location rural (ref = urban)	−0.024	− 0.134, 0.086	0.115	− 0.028, 0.258	0.045	− 0.179, 0.268
FP age/sex/rurality unknown (ref = < 50 years, female, urban known)	0.017	− 0.066, 0.101	0.043	− 0.065, 0.152	0.123	− 0.046, 0.293
Practice model (ref = FFS/FFS enhanced^c^)	**−0.069**	**− 0.108, − 0.029**	**−0.093**	**− 0.144, − 0.041**	−0.008	− 0.089, 0.072
Full-time equivalent (ref = less than full-time)	0.036	−0.005, 0.077	0.033	−0.021, 0.086	0.063	−0.021, 0.147
Mean % female patients	−0.001	−0.003, 0.001	− 0.002	−0.004, 0.001	− 0.001	−0.006, 0.003
Mean % patients with multimorbidity^c^	**0.006**	**0.005, 0.007**	**0.006**	**0.005, 0.008**	**0.004**	**0.002, 0.006**
***c. Linear Regression of Pandemic Mean Weekly Virtual Visits as a Proportion of Pandemic Mean Weekly Total Visits (N = 549)***						
**Pandemic Mean Weekly Virtual Visits as proportion of Mean Weekly Total Visits**	**Early 1st**^**a**^ **Wave** **Virtual Visits as proportion of Total Pandemic Visits**		**Mid-Late**^**a**^ **1st Wave** **Virtual Visits as proportion of Total Pandemic Visits**		**Early 2nd**^**a**^ **Wave** **Virtual Visits as proportion of Total Pandemic Visits**	
**Characteristics at Baseline**^**b**^	**Estimate**	**95% CI**	**Estimate**	**95% CI**	**Estimate**	**95% CI**
FP age > 50 (ref = < 50 years)	**0.058**	**0.006, 0.11**	**0.059**	**0.012, 0.106**	0.025	−0.022, 0.071
FP Sex Male (ref = female)	−0.023	− 0.086, 0.04	−0.021	− 0.078, 0.037	−0.002	− 0.059, 0.054
Practice location rural (ref = urban)	0.018	−0.132, 0.168	−0.093	− 0.23, 0.044	−0.062	− 0.197, 0.073
FP age/sex/rurality unknown (ref = < 50 years, female, urban known)	0	− 0.116, 0.117	0.005	− 0.102, 0.111	− 0.031	−0.138, 0.075
Practice model (ref = FFS/FFS enhanced^c^)	**0.13**	**0.076, 0.185**	**0.116**	**0.066, 0.166**	0.039	−0.01, 0.088
FTE (ref = less than full-time)	0.023	−0.036, 0.081	0.019	−0.035, 0.072	0.003	−0.05, 0.056
Mean % patients where sex = female^c^	**0.005**	**0.001, 0.008**	**0.004**	**0.002, 0.007**	**0.004**	**0.002, 0.007**
Mean % patients with multimorbidity^c^	**−0.008**	**−0.009, − 0.006**	**−0.006**	**− 0.008, − 0.005**	**−0.005**	**− 0.007, − 0.004**

^a^Pre-pandemic defined as 12 months prior to March 14, 2020, Early 1st Wave defined as March 14–May 10, 2020, Mid-Late 1st Wave defined as May 11–September 17, 2020, Early 2nd Wave defined as September 18–October 31, 2020

^b^Baseline is March 14, 2020

^c^FFS = Fee for service; Mean % sex = female is % of patients seen that were female, estimate is for additional 10% of females seen; Mean % patients with multimorbidity is % of patients seen that had multimorbidity, estimate is for additional 10% of patients having multimorbidity

The quantitative results demonstrated the role of payment models in the type of visits. This also arose as a theme in the qualitative study, providing some context to the quantitative results. FPs characterized this as “the inequity of payment models” created by having different physician compensation models in Ontario. FPs who were in capitation models received basic payments from the provincial government regardless of the overall decrease in total visits early in the pandemic; whereas, FPs in fee-for-service models were paid only when they had an encounter with a patient. This perceived inequity was highlighted by FPs in both types of models; “I’m fortunate, being in [a capitation model] because we still get paid; and so even when nobody was coming in we were essentially getting most of our pay … So doctors, who were on fee-for-service, for the first [few] months, are getting paid nothing.” (FP14, M, 63 years). This *inequity of payment models* subtheme intersects with the subtheme above concerning *initial volumes of visits dropped*. Fee for service FPs were more greatly affected by the overall drop in visit volumes during the early pandemic; the rise in virtual care did not completely offset the overall drop in visit volume. As well, while virtual visits were reimbursable as of March 14, 2020, the Ontario government was not able to accept these new virtual visit billing codes until May 1, 2020 [28, 29], again differentially impacting fee-for-service physicians who receive no capitation payments.

## Discussion

Using mixed methods, this study examined the experience of FPs during the early COVID-19 pandemic in London and Middlesex County. It was important to focus on this early pandemic period when much of FP care was virtual; this permitted us to see the impact that virtual care had on overall FP care before the late 2020 return to more typical in-person care. It was also important to conduct an in-depth regional examination of FP care, particularly a region like London-Middlesex, outside the Greater Toronto area with its more highly concentrated population. The use of ICES health administrative data provided population-level findings for the entire London and Middlesex County. The analysis and integration of quantitative and qualitative data over the same pandemic period in the same geographic region provides rich findings, not available through a solely quantitative inquiry.

The number of FPs seeing patients (in-person or virtually) decreased across the early pandemic. The initial volumes of patients seen decreased, driven by a decrease in in-person visits. Virtual visit volumes increased dramatically within 2 weeks of the start of the pandemic and with the implementation by the Ontario MOH of special billing codes that reimbursed FPs for virtual care including telephone and video [7]. This rapid move to virtual care was found in other Canadian jurisdictions [30] and international jurisdictions such as Australia [31, 32], the United States [32, 33], and China, Norway, Singapore, South Korea, Sweden, and the United Kingdom [32].

In interviews, FPs reported that a lack of PPE early in the pandemic resulted in the need to avoid in-person visits and their patients being reluctant to come to the office for fear of contracting COVID-19. FPs described using telephone visits almost entirely, which is consistent with other Canadian studies where preferred modalities were telephone, followed by email, and then video conferencing tools (e.g., Zoom, Skype) [1, 34–36] and international jurisdictions where telephone was often the primary means of care delivery [37]. Despite the availability of the OTN virtual care platform, the vast majority of the FPs interviewed did not use OTN, reporting it to be difficult for themselves and patients to use. This is consistent with previous literature where stakeholders criticize OTN for its lack of operability across platforms, inability to handle high volume of patients, and its complex user interface [38, 39]. Rural FPs reported connectivity issues with OTN and other video platforms due to lack of high-speed internet. Rural connectivity has been reported as a reason for telephone over video modalities in other jurisdictions [37].

By the early second wave of the pandemic (mid-September 2020), the total volume of FP visits returned to pre-pandemic volumes and in-person visits increased substantially, highlighting that FP offices were available to see patients and that in-person increased as the availability of PPE increased.

It was encouraging to see quantitatively that patients seen during the pandemic were older, with a higher percentage having multimorbidity, and more likely to be materially deprived; in other words, patients that needed care. This was consistent with Ontario-wide findings by Glazier et al. [5]. This also was consistent with what we heard during the interviews. Although FPs did not specifically use the language of multimorbidity, they told stories of specific patients such as vulnerable patients, patients who were older with mobility issues, and patients requiring follow-up for on-going chronic conditions such as diabetes. FPs noted that virtual care had improved access for these high-need patients during the pandemic when they could not be seen in-person or when they were reluctant to go into the FP’s office. Despite these encouraging findings, we cannot determine from our study the extent to which those with multimorbidity received all necessary care. Other studies have cited that chronic disease management and preventive care for those with chronic conditions suffered during the pandemic [37, 40]. In our study, while reporting better access because of virtual care, our FP participants cautioned that highly vulnerable patients such as those who were homeless and/or without access to a telephone were sometimes unable to access virtual care.

### Limitations

Our study was not able to determine reasons for the decrease in numbers of FPs practising during the early and middle waves of the pandemic; nor were we able to determine whether this change was usual fluctuation or particular to the pandemic. We reported on socio-demographic variables such as income and material deprivation; however, Ontario does not capture information on patients’ race or ethnicity, and sex is limited to male or female. Another limitation is that our interviews extended beyond the date at which our health administrative data was available, and months after the start of the pandemic. This could lead to recall bias; however, we think this risk was mitigated by the fact that the COVID-19 pandemic was a momentous event for everyone including family physicians. As such, we found family physicians, even those interviewed 7 months after the start of the pandemic, were able to remember vividly how and when they transitioned to virtual care. They recalled clearly patient stories, sometimes even quoting what patients had said to them about virtual care.

## Conclusion

The pandemic provided a disruption to the delivery of family physician care that brought about the removal of major barriers to providing virtual care in Ontario, Canada. Key among these barriers was the provision of compensation to FPs to offer virtual care through means other than the province-sanctioned OTN network, including by telephone. This study provides valuable messages to guide the future of virtual FP care including the importance of continued compensation for virtual care, the need for equitable FP payment models, and the need to attend to equity in access for highly vulnerable patients. Additionally, our study found that the move to virtual care during the pandemic was primarily a move to telephone care; therefore, when considering the success and future of virtual care, we must consider the modality of care delivery that is acceptable to both FPs and their patients.

## Supplementary Information

**Additional file 1: Appendix 1.** Administrative datasets used in study. **Appendix 2.** Interrupted Time Series for Family Physicians’ Mean Number of Weekly Visits (*N* = 549). **Appendix 3.** Virtual Family Physician Care during COVID-19: Interview Guide – Virtual Care Questions.

## Data Availability

The data set from this study is held securely in coded form at ICES. While data sharing agreements prohibit ICES from making the data set publicly available, access may be granted to those who meet prespecified criteria for confidential access, available at https://www.ices.on.ca/DAS. The full data set creation plan and underlying analytic code are available from the authors on request, understanding that the computer programs may rely on coding templates or macros that are unique to ICES and are therefore either inaccessible or may require modification.

## Abbreviations

COVID-19: Novel coronavirus 2019
CD: Census division
CGT: Constructivist Grounded Theory
FPs: Family physicians
FTE: Full-time equivalent
HA data: Health administrative data
IPDB: ICES Physician Database
OHIP: Ontario Health Insurance Program
OTN: Ontario Telemedicine Network
PPE: Personal protective equipment
