# Supplementary material for: Virtual family physician care during COVID-19: a mixed methods study using health administrative data and qualitative interviews
Source: BMC Prim Care. 2022 Nov 25;23:300. doi: 10.1186/s12875-022-01902-9 (PMC9700898; doi:10.1186/s12875-022-01902-9)
Supplement: Supplementary file 1 — Additional file 1: Appendix 1. Administrative datasets used in study. Appendix 2. Interrupted Time Series for Family Physicians’ Mean Number of Weekly Visits (N = 549). Appendix 3. Virtual Family Physician Care during COVID-19: Interview Guide – Virtual Care Questions. [file 12875_2022_1902_MOESM1_ESM.docx]

**Appendix 1. Administrative datasets used in study**

| **Dataset** | **Description** | **Variables** |
| --- | --- | --- |
| Canadian Institute for Health Information Discharge Abstract Database (DAD) | Contains administrative and clinical information on all admissions/discharges from acute care facilities in Ontario | Chronic conditions |
| Chronic Obstructive Pulmonary Disease (COPD) | Database containing patients identified as having COPD using a validated case algorithm | Chronic Obstructive Pulmonary Disease |
| Congestive Heart Failure (CHF) | Database containing patients identified as having CHF using a validated case algorithm | Congestive heart failure |
| Institute for Clinical Evaluative Sciences Physician Database (IPDB) | Yearly information on all physicians practicing in Ontario, including main specialty | Identification of family physicians  Physician age, sex, location (urban/rural) |
| National Ambulatory Care Reporting System (NACRS) | Contains patient-level data (demographic, diagnoses, procedures) for all visits made to hospital and community based ambulatory care centres (emergency departments, day surgery, dialysis, cancer care clinics) in Ontario | Chronic conditions |
| Ontario Asthma Dataset (ASTHMA) | Database containing patients identified as having asthma using a validated case algorithm | Asthma |
| Ontario Crohn’s and Colitis Cohort Dataset (OCCC) | Database containing patients identified as having Crohn’s or Colitis using a validated case algorithm | Inflammatory bowel disease |
| Ontario Dementia Database (DEMENTIA) | Database containing patients identified as having dementia using a validated case algorithm | Dementia |
| Ontario Diabetes dataset (ODD) | Database containing patients identified as having diabetes using a validated case algorithm | Diabetes |
| Ontario Drug Benefit Claims (ODB) | Contains information on public and private patient prescription claims for drug benefits | Chronic conditions |
| Ontario Health Insurance Plan Claims Database (OHIP) | Claims for all physician services provided to Ontario residents | Chronic conditions, physician visits |
| Ontario HIV Dataset (HIV) | Database containing patients identified as having HIV using a validated case algorithm | HIV |
| Ontario Hypertension Dataset (HYPER) | Database containing patients identified as having hypertension using a validated case algorithm | Hypertension |
| Ontario Marginalization Index (ONMARG) | Data tool that combines a wide range of demographic indicators into four distinct dimensions of marginalization. The material deprivation dimension was used for this study. | Material deprivation quintile |
| Ontario Mental Health Reporting System (OMHRS) | Contains data on adult designated inpatient mental health beds (incl. general, provincial psychiatric, and specialty psychiatric facilities) using the Resident Assessment Instrument - Mental Health | Mood disorder |
| Ontario Rheumatoid Arthritis Database (ORAD) | Database containing patients identified as having rheumatoid arthritis using a validated case algorithm | Arthritis |
| Primary Care Population (PCPOP) | Contains basic demographic information and Primary Care Rostering (which physician/group and family health team a patient is enrolled with) | Physician practice model |
| Registered Persons Database (RPDB) | A population-based registry that contains demographic information for all residents of Ontario who have registered for health insurance. | Patient age, sex, income quintile |
| Same Day Surgery Database (SDS) | Information on same day surgeries performed in Ontario | Chronic conditions |

**Appendix 2. Interrupted Time Series for Family Physicians' Mean Number of Weekly Visits (N = 549)**

|  | **Pre-pandemic level** | | **Trend** | | **Pandemic level** | | **Trend after** | |
| --- | --- | --- | --- | --- | --- | --- | --- | --- |
|  | **Estimate, 95% CI** | **p-value** | **Estimate, 95% CI** | **p-value** | **Estimate, 95% CI** | **p-value** | **Estimate, 95% CI** | **p-value** |
| Visits overall | 81.27  (76.19, 86.34) | <.0001 | 0  (-0.17, 0.17) | 0.9978 | -25.01  (-33.07, -16.95) | <.0001 | 0.77  (0.4, 1.14) | 0.0001 |
| Virtual visits | -0.01  (-1.62, 1.6) | 0.9875 | 0  (-0.05, 0.06) | 0.8667 | 33.52  (30.97, 36.07) | <.0001 | -0.14  (-0.25, -0.02) | 0.0238 |
| In-person visits | 81.59  (75.73, 87.45) | <.0001 | -0.02  (-0.21, 0.17) | 0.8301 | -57.2  (-66.34, -48.07) | <.0001 | 0.89  (0.47, 1.32) | <.0001 |

**Appendix 3. Virtual Family Physician Care during COVID-19: Interview Guide – Virtual Care Questions**

1. I would like you to go back to the world prior to March 14 [2020 when Ontario Ministry of Health instituted virtual visit billing codes] and prior to public health measures concerning COVID-19. Can you spend a few minutes talking about how you delivered care pre-COVID-19?
2. So now, thinking around March 14 and moving to virtual visits, can you share the early days? What was your experience of getting everything in place for virtual visits?
3. So now you have virtual visits up and running - can you share the process of doing virtual visits?
4. Were there personal or family issues that made it challenging for you to provide virtual visits?
5. Can you share with me, looking back, what lessons you learned?
6. If you were writing best practice guidelines for virtual care and had the ear of the Ministry [of Health], what would you tell them we need to do next time we face a pandemic?
